# How AI Shapes the Future Landscape of Sustainable Building Design With Climate Change Challenges?

**DOI:** 10.1002/advs.202523238

**Published:** 2026-01-31

**Authors:** Pengyuan Shen, Xiaoni Gao, Shuai Lu, Yi Zhang, Xing Zheng, Matthaios Santamouris

**Affiliations:** ^1^ Institute of Future Human Habitats Shenzhen International Graduate School Tsinghua University Shenzhen China; ^2^ Department of Architecture and Civil Engineering City University of Hong Kong Hong Kong SAR China; ^3^ School of Built Environment University of New South Wales Sydney Australia

**Keywords:** artificial intelligence, building simulation, climate change, foundation model, sustainable building

## Abstract

Faced with climate change challenges, artificial intelligence (AI) is redefining the way of sustainable building design. In this work, how AI technologies, including foundation models and generative systems, are reshaping architectural practice in responding to climate change is discussed. We explored how large language models, multimodal systems, and automated design generation have evolved from traditional computational methods, and the transformative potential of these novel approaches, especially when dealing with climate change challenges. While AI holds powerful tools for sustainable architecture, we argue that the successful implementation of those tools requires careful integration of technical capabilities, practice frameworks, and regulatory considerations. To advance AI‐driven sustainable building design while providing effective future climate response, research priorities and policy recommendations are put forward in this study.

## Graphical abstract

This review examines how artificial intelligence reshapes sustainable building design faced with climate change challenges. The authors synthesize existing studies to demonstrate AI's transformative potential across design lifecycle phases from climate‐aware form generation to performance optimization. They propose the AI‐Climate‐Building Integration (ACBI) framework, establishing technical, climate response, and governance pillars essential for deploying AI tools effectively in climate‐responsive architecture.

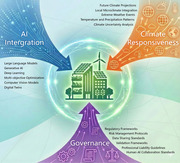

## 1 Introduction

The urgency of future sustainable building design is imminent when faced with the challenges imposed by intensifying climate change and urbanization trends. As buildings contribute to 35% of the world's total energy consumption and around 40% of greenhouse gas emissions [1], the demand for climate‐responsive architecture is more critical than ever. Recent advances in Artificial Intelligence (AI), such as machine learning [2]. and large language models (LLMs) [3], are redefining how we address the pressing need for low‐carbon, climate‐adaptive building solutions and are initiating a paradigm shift that extends far beyond traditional computational design. While earlier computational approaches contributed significantly through parametric modeling, performance simulation, and rule‐based optimization, emerging AI systems are driving a deeper transformation by enabling more adaptive, data‐driven, collaborative, and integrated design processes [4]. Similar to AI‐driven revolutions in protein structure prediction [5, 6], mechanical metamaterial design [7, 8], and drug molecule discovery [9, 10], breakthroughs in generative AI and foundation models will dramatically accelerate the evolution toward intelligent design workflows, with growing evidence of their ability to learn climate‐responsive design intent across future scenarios and allow more robust solutions [11, 12]. These systems process high‐dimensional environmental and technical data to address climate‐induced complexity [13, 14]. Holistic simulation and consideration of sophisticated building‐climate interactions across multiple scales, such as form‐finding, material selection, climate adaptive strategy proposition, and construction, can also be made possible via AI‐driven approaches. These capabilities, coupled with generative design, iterative optimization, and human‐AI collaboration, have the potential to produce climate‐adaptive solutions that would have been inconceivable through conventional methods alone. Specifically, modern AI systems can process multiple design variations through thousands of analyses while integrating contextual knowledge to meet performance standards across different climate scenarios. Nevertheless, questions remain in the state of the art regarding technical robustness, real‐world integration, future climate responsiveness, and alignment with architectural values and regulatory requirements of the AI‐driven solutions in architectural practice.

**FIGURE 1:**
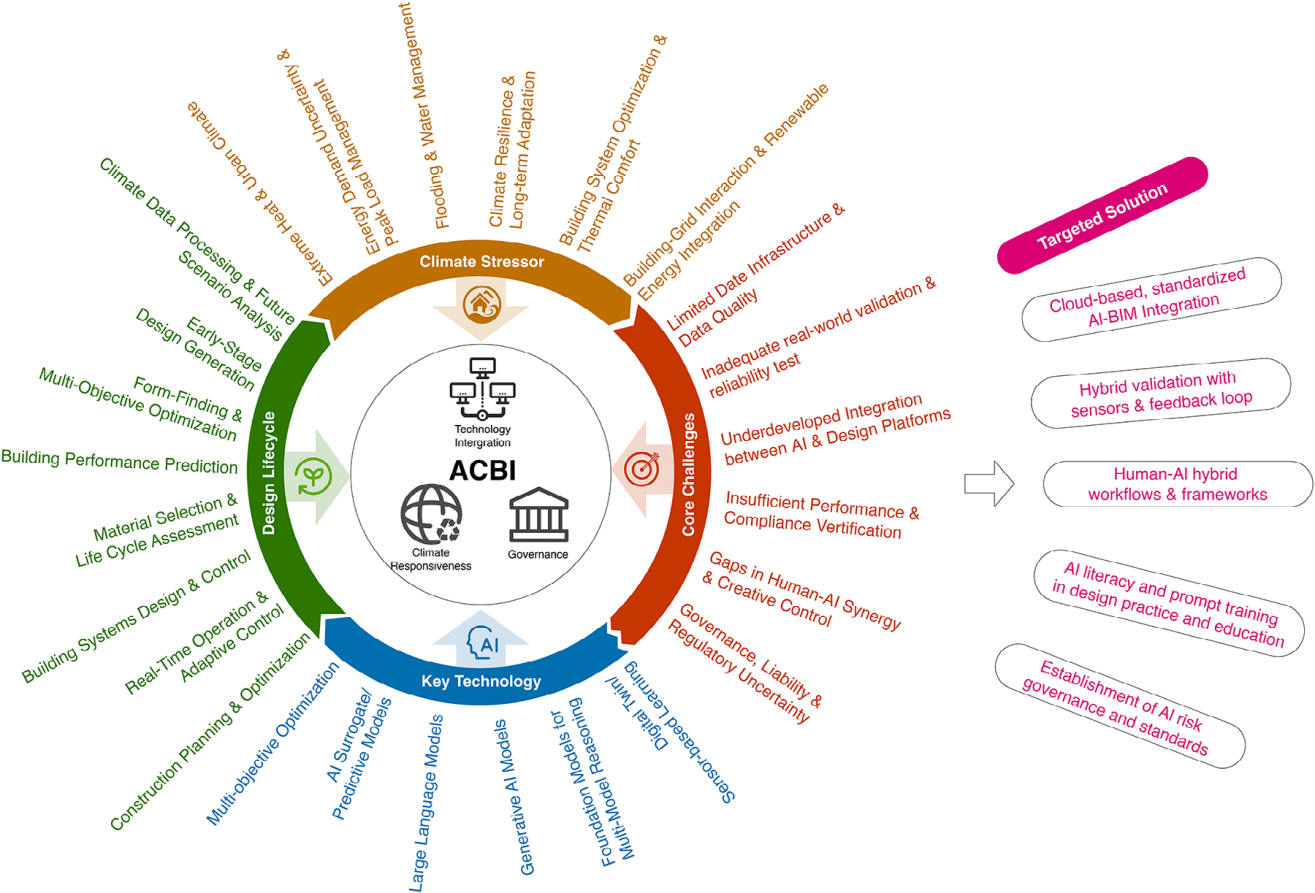
The overall framework of this paper. It ties together key AI technologies, design lifecycle phases, climate stressors, core challenges, potential future solutions, as well as our ACBI framework.

In the context of climate change, this work tries to interrogate the question of how AI is going to change sustainable building design. Drawing on a comprehensive literature analysis, we structure our perspective around four levels of inquiry, which is shown in Figure 1: the current state of AI applications in climate‐responsive architecture, the transformative effects of generative AI and foundation models in the design process and in response to the climate stressors, the frameworks for the deployment of these technologies in practice, and future directions and challenges in AI‐driven sustainable architecture. Recent studies in AI for the built environment have made valuable advances and demonstrated impressive progress across several key domains, such as early‐stage performance optimization and form exploration, building operations and smart grids, and climate‐adaptive building design. Synthesizing upon them, our paper provides a broader integration across these domains through the lens of foundation models, multi‐modal reasoning, and long‐term climate adaptability. Specifically, our synthesis shows that foundation models (e.g., GPT‐4o, Stable Diffusion 3) offer general‐purpose capabilities that are more flexible in addressing climate stressors compared to task‐specific models. We also highlight the importance of multi‐modal reasoning in the AI era to interpret and generate across different data forms: textual, visual, spatial, and quantitative modalities. Moreover, we argue that under the current intensifying climate change, future climate scenarios should serve as a design baseline, not a peripheral consideration, and systematically explore how AI can address the resulting uncertainty and complexity.

By critically synthesizing individual studies, we propose a structured, systemic conceptual framework called the AI‐Climate‐Building Integration Framework (ACBI), in order to deploy the powerful tools offered by AI to support sustainable building goals in an effective way. This framework includes three interdependent pillars that determine the success of implementation: (1) Technical Integration Pillar: the creation of dynamic layers of information coupling, allowing two‐way data flow between AI systems, the creation of information models, and real‐time environmental sensors; (2) Climate Response Pillar: the development of systems that are able to process future climate projections, assess environmental performance in the face of dilemmas and to develop adaptive design strategies; and (3) Governance Pillar: the creation of risk management protocols, standards for sharing data, and regulatory strategies that ensure responsible AI deployment and the facilitation of innovation. The ACBI Framework goes beyond the descriptive taxonomies by suggesting testable relationships. The quality of technical integration has a direct impact on the accuracy of climate‐responsive forecasts, and the accuracy of the latter determines the effectiveness of the governance measures. This causal chain provides the basis for future empirical validation and cross‐study comparison.

It should be acknowledged that technological sophistication alone is insufficient. The value of AI‐driven solutions ultimately lies in their ability to interface with real‐world constraints and respond meaningfully to the complexities introduced by climate change. Moreover, beyond technical optimization, AI also has a role to play in supporting creative design processes and aligning with climate adaptation strategies. We believe the time has come for the architectural profession to understand how to best leverage AI as climate challenges become increasingly complex and urgent for the building sector.

## 2 Review Methodology

This review used a structured narrative approach in order to synthesize the literature on the application of AI in the field of sustainable building design. We searched Web of Science, Scopus, and Google Scholar using combinations of keywords such as ‘artificial intelligence’ OR ‘machine learning’ OR ‘deep learning’ OR ‘large language model’ AND ‘sustainable building’ OR ‘green building’ OR ‘climate‐responsive architecture’ OR ‘building energy’ OR ‘building performance optimization’. The search was conducted in publications from January 2010 to October 2024, and a focus was put on the post‐2020 publications to reflect the latest progress in the world of foundation models and generative AI. Inclusion criteria included: (1) peer‐reviewed journal articles, conference papers, and authoritative technical reports, (2) studies that reported on the use of AI in building design, performance prediction, or climate adaptation, and (3) English‐language publications. Exclusion criteria were: (1) study only on building operation and not covering the design implications; (2) purely theoretical AI papers, not covering building domain application; (3) duplicate publications.

**FIGURE 2:**
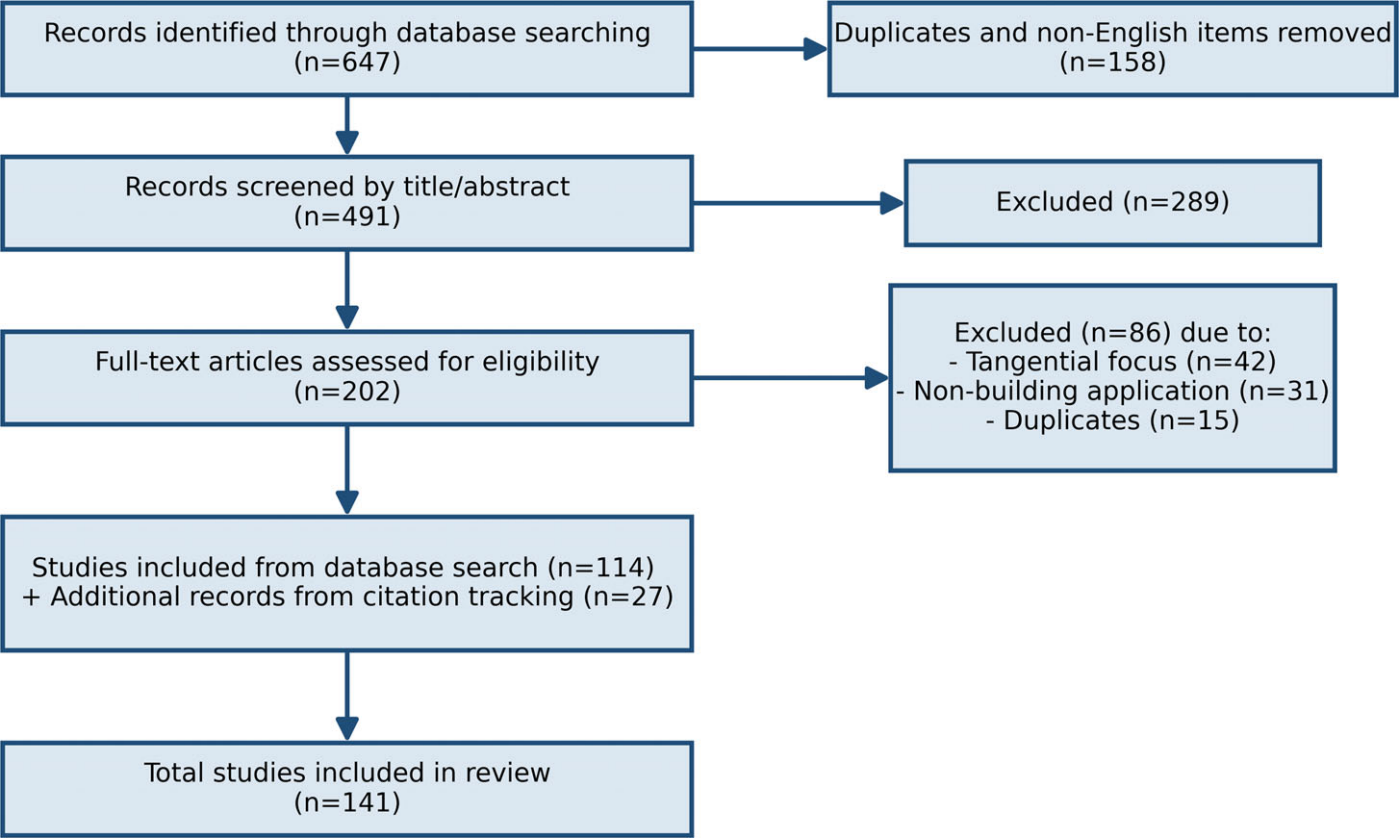
Literature screening process and flow diagram.

Our searches came up with 647 records at first. After the removal of duplicates (n = 156) and screening of the titles and abstracts for relevance (excluding n = 289), a total of 202 full‐text articles were assessed. Of these 116 fulfilled the inclusion criteria and formed the basis of this review. While this work is more of a narrative review than a systematic review, this approach allows for transparent coverage of the fast‐developing intersection of AI and sustainable building design. The flow diagram for the literature screening process of the present review work is plotted in Figure 2.

## 3 Emergence of AI for Sustainable Building Design

The concept of “sustainable development” first formed in the 1970s when the first energy crisis took place, which was later consolidated by the Brundtland Report in 1987 [15]. Later, the emergence of the concept “sustainable building” can be dated back to the 1990s when the United Kingdom and the United States began to introduce green building standards, though the notion of sustainable building and green building slightly differs nowadays [16]. Content‐wise, sustainable architecture has arisen from the confluence of empirical knowledge and scientific understanding of building physics [17]. Passive strategies and efficient active system integration are the main focus of traditional sustainable design approaches. The foundation of sustainable architecture encapsulates passive design strategies, including but not limited to orientation, form, and envelope design, to maximize natural ventilation, daylighting, and thermal performance [18]. Solar shading, thermal mass utilization (a strategy closely related to demand response and building‐grid interaction), and natural ventilation systems have been shown to be able to effectively improve indoor environmental quality and occupant comfort, as well as reduce building energy consumption [19]. However, they can be less effective in some climate zones and become increasingly unpredictable under a changing climate [20, 21].

Structured systems to quantify and improve building performance have emerged in the form of energy efficiency guidelines and frameworks. Popularly referenced building performance codes like BREEAM, LEED, CASBEE, etc., have set up metrics and benchmarks for building energy consumption, indoor and outdoor multi‐sensory comfort, and environmental impact [22]. They assess buildings’ sustainable performance based on existing simulation tools and post‐occupancy evaluation. Building energy simulation software allows designers to predict energy consumption and thermal behavior [23], while environmental assessment tools can assess broader sustainability metrics [24]. These frameworks were driving industry transformation and have played a critical role in promoting measurable and performance‐driven sustainable architecture.

However, these frameworks are fundamentally grounded in static models, historical climate data, and isolated design workflows, limiting their capability of addressing dynamic climate challenges. Most building performance simulations rely on complex, physics‐based engines that are computationally intensive and often difficult to calibrate, particularly in early‐stage design. While effective in past applications, such tools are increasingly challenged by the rising uncertainty of future climate conditions, growing demand for resilience, and accelerated timelines imposed by climate emergencies. Moreover, standardized weather files based on past climate data may fail to address climate uncertainty and capture future extremes [25], and conventional tools lack the ability to rapidly test adaptive solutions under evolving scenarios, creating a potential mismatch between predicted and actual building performance [26]. In addition, climate change introduces multidimensional design pressures that extend beyond traditional building performance codes, such as flood mitigation, urban overheating, disaster risk reduction, and broader ecological resilience [21]. These priorities require the processing of diverse and dynamic data sources and coordinating decisions across multiple scales and design phases. While traditional strategies like solar shading, thermal massing, and envelope tuning remain essential, their performance is no longer guaranteed in future contexts, and thus must be reconsidered in earlier, more integrated stages of the design process. Moreover, the integration of new energy and environmental technologies, such as building‐integrated photovoltaics (BIPV), high‐performance insulation, and phase‐change materials, is becoming increasingly critical for achieving climate‐responsive built environments. However, the complexity introduced by these technologies further exposes the limitations of traditional design methods, which struggle to accommodate the dynamic, interconnected demands of future‐oriented sustainable architecture.

In order to overcome the restrictions of traditional methods, the application of AI in architectural design is a crucial step from the use of early computational methods to contemporary AI systems. Computational design started with computer‐aided design (CAD) in the 1960s [27], parametric modeling [28], and culminated in the current AI‐driven approaches that are capable of autonomously generating and evaluating design solutions [29]. Currently, Large language models (LLMs) [30] and computer vision models [31] have emerged as powerful foundation models in architectural design. These models can be trained on massive architectural domain knowledge as well as visual data and understand complex design requirements in order to generate contextually appropriate solutions. LLMs such as GPT‐4o and GPT‑5.2, among the most representative current AI systems, can interpret architectural briefs, generate design specifications, and even suggest sustainable design strategies given the climate conditions, though detailed and tailor‐made design schemes are still limited in non‐fine‐tuned LLMs [32, 33]. The recent emergence of open‐source foundation models such as DeepSeek [34], which is particularly apt at reasoning and multi‐modal input, further amplifies the scalability and possibility of LLM application in domain‐specific architectural contexts.

The computer vision models, on the other hand, are able to analyze existing architectural precedents and derive design principles that respond to particular environmental challenges, for example, daylighting‐driven architectural design [35]. Text‐to‐image [36]. and text‐to‐3D [37]. generation are also revolutionary advances in architectural design tools. Models like DALL·E, Midjourney, and the most current Stable Diffusion 3 can take textual descriptions and produce architectural visualizations. Advanced text‐to‐3D models are capable of generating building geometries from natural language descriptions [38]. Deep learning algorithms such as U‐net can support the automated generation of building layout graph [34, 39]. Recently, Sora, a video‐based foundation model, has shown the potential to simulate spatial experiences and occupant interactions over time. Yet, geometric accuracy and physical feasibility limitations still currently preclude automatic operation and demand careful human oversight [40].

The above models have the crucial ability to process multimodal data (e.g., text, numeric, geometry), including future climate scenarios, to understand regulatory constraints, to simulate performance, and to enable interactive generation and selection of optimal alternatives. For example, AI can parse climate‐responsive design briefs to create material specifications that adhere to specific carbon reduction targets in particular regions under future climate extremes, thus linking algorithmic design with effective architectural strategies. These capabilities are particularly useful in the early design phase, where options for sustainable design can be rapidly explored, and their possible environmental impact can be evaluated. Collectively, these capabilities provide the foundation for an expanded, human‐AI collaborative design workflow by linking algorithmic synthesis with human creativity and performance intelligence.

**FIGURE 3:**
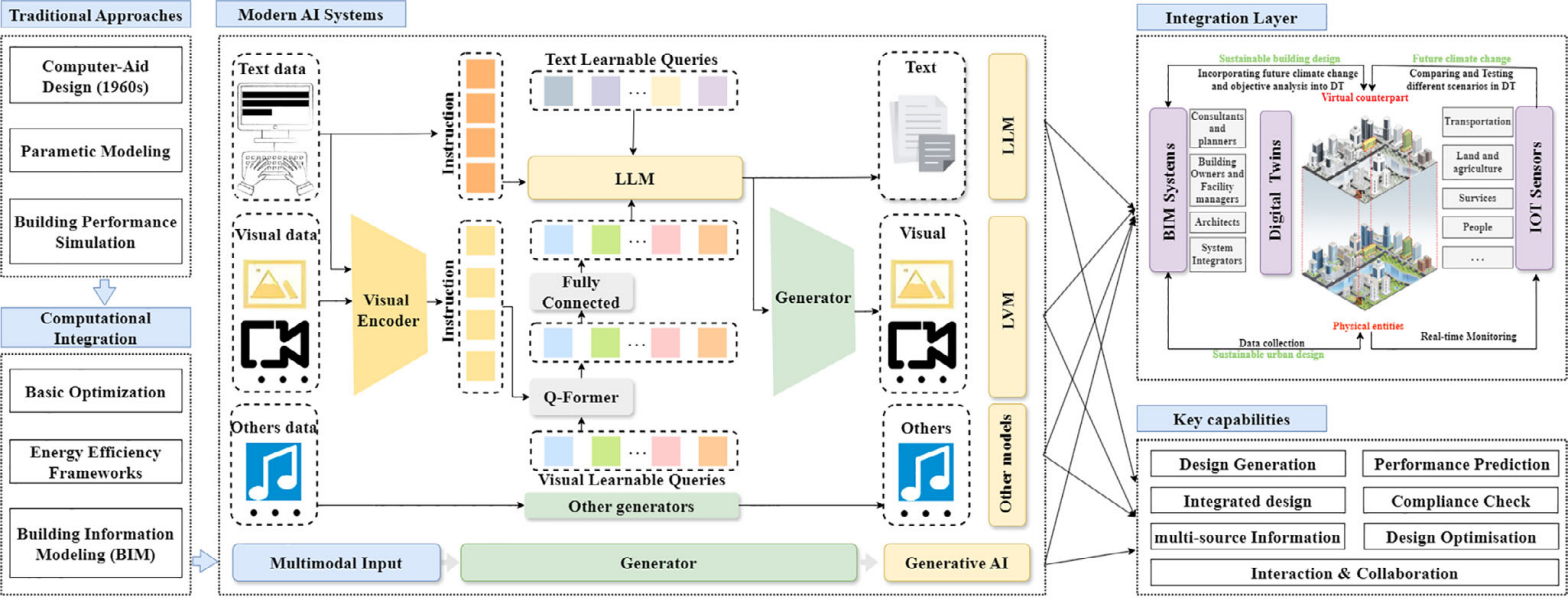
Historical development and AI emergence for sustainable building design methods. Traditional approaches: People initiated using computers in assisting architectural design by introducing parametric modeling and building performance simulation methods to sustainable architecture during the 1960s. Computational Integration: Computational technology partnered with sustainable architecture after 2000 to achieve optimization of basic operations and energy efficiency frameworks through Building Information Modeling (BIM). Modern AI Systems: Modern AI systems emerged in the early 2020s, marked by the rise of foundation models such as Stable Diffusion (2022) and ChatGPT (2022), and have since progressed to today's cutting‐edge multimodal models, including GPT‐4o, Sora, and Stable Diffusion 3. These advanced systems are increasingly popular in sustainable architectural design, as they can generate climate‐adaptive solutions using various data modalities by leveraging Large Language Models (LLMs), Large Vision Models (LVMs), and other AI models. Integration Layer: The integration layer maintains communication between AI‐created sustainable designs and upcoming climate limitations. The integration framework includes three components: (1) systems like BIM that can deliver static information about geometry and attributes and historical data; (2) IoT Systems that provide real‐time environmental data; (3) digital twins that unite static (BIM) and dynamic (IoT) data for interactive assessment. The integrated system helps decision making for operational needs while also optimizing energy efficiency by considering future climate predictions. Key Capabilities: The main abilities of generative AI systems consist of form‐finding & optimization, together with performance prediction and climate‐responsive analysis.

However, there are also integration challenges with current practices when it comes to AI adoption. AI tools are showing promise, but incorporating them into existing architectural workflows is complicated. Those challenges include compatibility with Building Information Modeling (BIM) systems, validation of the AI‐generated designs with building codes, and standardization of protocols for AI‐human interaction and collaboration [41]. At the same time, the architectural profession also needs to deal with the changing role of the architect in an era where design generation and evaluation are being supported by AI techniques and tools, which are shown in Figure 3. This shift necessitates a rethinking of traditional design processes and the development of new frameworks that can seamlessly integrate AI into architectural practice. Practical implementation barriers, including data availability and workflow integration, will be addressed in later discussions in Sections 5 and 6.

## 4 AI‐Driven Design Transformation

### 4.1 AI‐Empowered Climate Change Awareness in Design and Responsive Strategies

One of the most critical contributions of AI to sustainable building design lies in its capacity to enhance awareness of future climate conditions and enable more resilient design strategies. While future climatic data is available through methods like deterministic models, AI plays a crucial role in processing, interpreting, and extracting actionable insights from such complex datasets [42, 43]. Contemporary AI models can identify key trends, such as shifts in temperature, precipitation, and wind patterns, which are then used to inform early‐stage design decisions [20, 44].

In particular, AI‐processed climate variables are converted into constructive design decisions through different pathways. Analysis of temperature patterns, especially cooling and heating degree days, directly informs envelope design, including thermal mass, glazing ratios, and insulation levels. AI models assessing future diurnal temperature fluctuations can optimize thermal mass configurations to buffer indoor temperatures through effective heat storage and release [21]. Precipitation pattern changes, such as the intensity and frequency of extreme rainfall events, inform decisions of roof drainage capacity, specifications for foundation waterproofing, and site grading. AI analysis of projected precipitation extremes allows sizing of stormwater management systems and also affects the choice between permeable and impermeable surface materials. Wind pattern changes influence the viability of natural ventilation strategy, building orientation optimization, and structural wind load computations. Machine learning algorithms handling wind rose projections can be a means to optimize building form and opening configuration to maximize passive cooling potential whilst maintaining structural resilience in the event of intensifying storm events. These climate‐to‐design linkages are shown in Table 1, which describes how climate data flows through digital twin systems to inform certain design parameters.

**TABLE 1:** Climate variable to architectural design strategy mapping.

Climate variable	AI processing capability	Design strategy output	Building system affected
Temperature shifts (mean, extremes)	Trend detection, anomaly identification	Thermal mass configuration, glazing ratios	Envelope, HVAC sizing
Precipitation changes (intensity, frequency)	Extreme event prediction	Drainage capacity, waterproofing specs	Site design, roof systems
Wind pattern shifts	Directional analysis, speed projections	Orientation optimization, opening design	Natural ventilation, structure
Humidity changes	Seasonal pattern analysis	Vapor barrier placement, material selection	Envelope, material systems

Machine learning algorithms can be adopted to downscale global climate models (GCM) to building‐relevant spatial and temporal resolutions, identifying local climate shifts that inform design decisions [14, 42]. AI models can process diverse climate projection datasets to extract actionable insights about temperature shifts, precipitation patterns, and extreme weather events [45], enabling early‐stage design decisions grounded in projection rather than historical conditions. This capability proves particularly critical for long‐lived infrastructure, where design decisions made today must perform across decades of climate change. Recent implementations demonstrate that GAN‐based urban form generation integrated with microclimate simulation can achieve 1.8°C nighttime temperature reductions through optimized design, while poorly designed high‐density developments showed temperature increases of 2.3°C [46]. Machine learning applications in urban regeneration have documented SUHII reductions of 0.94°C in summer and 0.54°C in winter following targeted interventions, including 19.46% vegetation cover increases and 3.09% albedo improvements [47]. These quantifiable outcomes validate AI's role in designing thermally resilient urban environments, particularly as heat stress becomes a defining challenge for cities worldwide. When it comes to flooding prediction and informing building design, AI‐powered forecasting transforms flood preparedness from reactive response to proactive adaptation. Machine learning has extended reliable flood forecasts from zero to five days globally, with Google's Flood Hub now providing seven‐day advance forecasts across 80+ countries covering 460 million people [48]. Critically, AI‐based systems have improved forecast accuracy in data‐scarce and underdeveloped regions to levels comparable with Europe, addressing longstanding inequities in climate adaptation capacity. This enables building designers and urban planners to incorporate site‐specific flood risk into early design decisions, from elevation strategies to material selection and evacuation planning.

In parallel, the coupling of machine learning algorithms with building physics models has made it possible to simulate and optimize building performance across a wide range of projected climate scenarios [20, 25, 49]. This allows for a more nuanced understanding of how buildings will perform under future conditions, moving beyond historical weather files and static assumptions. By identifying subtle changes between historical and future climatic patterns, AI systems can help architects tailor climate‐adaptive strategies that are better suited to local conditions, as we have discussed in earlier sections. Moreover, the incorporation of uncertainty analysis into design workflows potentially enables the exploration of multiple climate scenarios, supporting the development of robust and flexible building solutions. With access to more accurate future weather data, such optimizations of building climate‐adaptive systems through AI‐driven approaches can move us one step closer to more efficient and sustainable architecture.

These capabilities also allow AI to enhance the real‐world effectiveness of climate‐adaptive strategies by optimizing the operation of responsive elements in real‐time. Contemporary algorithms can manage complex responsive systems such as dynamic shading and adaptive ventilation by learning from occupant behavior and changing environmental conditions [50]. Integrated with sensor networks, IoT devices, and building management systems, AI approaches enable continuous monitoring of how buildings respond to external climate variations [51, 52]. As a result, climate‐responsive building elements can be dynamically adjusted to improve energy efficiency, thermal comfort, and overall environmental performance [53], thereby maximizing the impact of the intended climate‐adaptive design. Data generated in this process, in return provides valuable feedback for future sustainable design. Collectively, these advances position AI as a critical enabler of climate‐aware architecture, capable of supporting both robust long‐term planning and real‐time adaptability under conditions of increasing climate uncertainty.

### 4.2 Design Generation Integrated with Multi‐Source Information Processing

AI's capability of processing diverse, multi‐source information is also critical to sustainable building design, especially under the current broad range of multidimensional design pressures introduced by climate change, such as flood mitigation, urban overheating, and disaster risk reduction. The complexity of information across diverse and dynamic sources is beyond the capability of individual designers or engineers, and this is where AI's ability comes into play.

**FIGURE 4:**
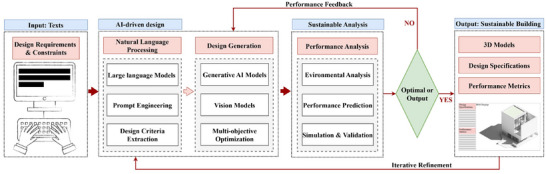
AI‐driven design generation pipeline for sustainable buildings. Input Texts: Design specifications for sustainability, along with their boundaries, were entered into the system as input texts. AI‐driven Design: The first phase of AI‐driven Design involves LLMs analyzing design requirements to obtain essential design criteria and parameters. Generation of sustainable building visual models through multi‐objective optimization occurs by processing the criteria and parameters through generative AI systems. Sustainability Analysis: The AI‐enabled sustainable building models then go through sustainability evaluation that includes environmental assessments, performance predictions, and simulation testing. Outputs: Sustainable building designs go through performance tests to determine their effectiveness under projected climate‐change conditions and their related designer specifications. AI‐driven design processes will operate with iterative refinement that uses performance feedback until they accomplish certain compliance or objectives. The final outcome will consist of a validated sustainable building model.

The emergence of LLM in recent years has unlocked unprecedented possibilities to translate design requirements into executable architectural specifications [33, 54]. These models can interpret complex architecture project briefs, environmental regulations, and performance requirements, and generate design specifications incorporating sustainable strategies [55]. Based on the understanding of contextual requirements, LLM also exhibits a remarkable ability for contextual reasoning and multi‐objective balancing, enabling it to suggest coherent, sustainable, and diverse design options that align with both performance targets and architectural intent, thus narrowing the gap between human design aspirations and computational generation. Given that model fine‐tuning still consumes huge computational costs and entails abstruse professional knowledge [56, 57], prompt engineering has been a key ingredient for architectural design, allowing for finely tuned AI‐generated deliverables at the current stage of AI development [58]. Architects will be able to explore design variations in generative AI systems by structuring queries that align their design work with sustainability goals and performance requirements. Recent research has shown that well‐designed prompt strategies can improve building energy performance [59]. However, comparative studies, both within the building domain [60, 61] and more broadly across AI research [62], have shown that even under identical prompts, different LLMs can yield substantially different outputs. These differences exist not only between general‐purpose models (e.g., GPT‐4o vs. DeepSeek), but also between general‐purpose and domain‐specific fine‐tuned models, the latter often exhibiting stronger contextual alignment and technical accuracy. This suggests that while prompt engineering remains critical at the current stage, future research may benefit from hybrid approaches combining optimized prompting and domain‐specific, knowledge‐enhanced model adaptation for more robust and verifiable outputs. This is especially valuable during early‐stage design, where rapid iteration and flexible exploration are critical. The plausible pipeline of the suggested workflow is proposed and illustrated in Figure 4.

Beyond textual data, text‐to‐design and image‐to‐design generation pipelines are another important step forward in supporting sustainable building design. They can transform textual descriptions into 3D architectural models, facilitating further environmental analysis and performance metrics [63, 64]. AI models capable of interpreting professional language and generating geometric structures have paved the way for a more comprehensive, integrated approach to sustainable building design. However, existing implementations still struggle to guarantee physical feasibility and regulatory compliance with generated designs. That is to say, though promising, the pressing imperative to develop robust implementation frameworks to bridge the gap between technological potential and real‐world application is the utmost challenge faced by emerging AI capabilities. In addition, AI enables the concurrent generation of comprehensive performance evaluation in parallel with design, the lagging of which is inevitable with traditional methods. By processing vast amounts of historical building performance data alongside changing climate patterns, deep learning algorithms can now predict building behavior with greater accuracy, speed, and depth [65]. In controlled studies, these models have shown particular success in forecasting energy consumption, thermal comfort, and daylight utilization [66, 67, 68], making performance evaluation an increasingly accessible and inseparable component of the early design process. As more diverse data becomes available, these capabilities can also be extended to addressing new challenges introduced by climate change, such as flood mitigation and urban overheat [69].

### 4.3 Automated Multi‐Scheme Generation, Optimization, and Interactive Co‐Design

Building on the understanding of climate scenarios and the effective processing of diverse information, AI‐driven approaches enable efficient design generation, optimization, and interactive co‐design, responding to dynamic climate conditions and diverse building performance requirements. Architectural form‐finding and environmental performance evaluation processes are now going through an evolution that is fundamentally transformed through AI‐driven approaches and optimization procedures. Now, AI‐driven systems have the potential to explore thousands of design alternatives while also optimizing for climate responsiveness through advanced generative algorithms [11, 70], addressing computation challenges that traditional methods struggled with. These models can understand complex relationships between geometry, orientation, and environmental performance, creating solutions that dynamically respond to local climate conditions [71]. Recent applications have shown advantages in building energy performance using AI‐optimized form generation over traditional design approaches [72]. Commonly involved optimization objectives included energy consumption and thermal comfort, daylighting, material usage and embodied carbon, and construction resource efficiency. Rather than relying on specific weightings, most studies adopt a Pareto optimization approach, which seeks a set of non‐dominated solutions where no objective can be further improved without compromising another. This approach allows designers to balance competing requirements, ensuring optimal environmental performance while respecting practical constraints [73, 74, 75].

By extending beyond traditional parametric approaches, AI design space exploration provides architects and engineers the opportunity to explore novel solutions that may otherwise be underexplored. By turning to machine learning algorithms, successful sustainable designs can be identified, and innovative alternatives that break conventional wisdom while meeting performance requirements can be generated [49, 76]. Such capability is especially useful in dealing with the challenges of climate change, where classic design solutions may not suffice. AI design scheme synthesis has expanded the potential for sustainable architecture, as well as posing questions of creative (artistic) control and design validation [77, 78]. Although AI systems can produce design variations optimized for environmental performance, the integration of these solutions with architectural schemes and cultural context is still a challenge [79]. When designers need to tackle complex sustainability requirements, the balance of human design judgment and AI‐driven optimization becomes critical in order to avoid outcomes that are technically efficient but contextually inappropriate or lacking in aesthetic and experiential qualities.

### 4.4 Integrated Design across Form, Material, Systems, and Construction

In traditional preliminary design, each step was done sequentially, without the ability to precisely assess performance or find the optimal solution, because the later stages were unknown. Under the demand for climate responsiveness, the deliverables of this approach can be suboptimal. AI enables integrated design by allowing all aspects, including form, materials, systems, and construction, to be considered together from the beginning, ensuring the best climate‐adaptive strategies are identified early on. AI also has the capability to select materials considering environmental impact, performance requirements, and life cycle factors [80]. Smart material selection algorithms can evaluate large databases of material properties, environmental data, and performance metrics, and recommend the best solutions for a given climate context [81]. Such algorithms can facilitate the specification of sustainable building components to higher precision, predicting their material behavior in different environmental conditions [82, 83].

Moreover, AI facilitates more targeted and integrated design of building systems that align with climate adaptation goals by providing access to detailed energy use patterns and predictive system responses [84]. By combining deep learning with complex interactions between HVAC, lighting, and other building systems, AI makes it possible to maximize building performance while minimizing energy consumption [85, 86, 87]. By learning from operational data, these models can predict system behavior under various conditions and can be used for proactive optimization strategies that maintain a high‐quality indoor environment while reducing system energy consumption by up to 30% [88, 89, 90]. Advanced neural networks further support dynamic adjustments based on occupancy patterns, weather shifts, and energy demand, making performance optimization continuous rather than static. As a result, optimal system control and precise energy management strategies become more readily achievable, feeding back into the design phase to inform system selection and integration decisions that enhance both energy efficiency and indoor environmental quality [91, 92]. AI's capability to predict not only overall energy consumption but also specific end‐use patterns enables the development of more targeted and efficient system design strategies [93].

AI also helps optimize the construction process to purvey sustainability considerations beyond the traditional design phase. Construction sequences, material logistics, and the utilization of resources can be optimized by machine learning algorithms, leading to less waste and lower environmental impact during construction [94, 95]. They offer the ability to predict and prevent construction challenges while facilitating the execution of sustainable design intentions into built reality. By incorporating AI‐driven analysis, lifecycle analysis (LCA) can be more precise in a way that allows building environmental impacts to be better evaluated over the entire life cycle [96]. Complex LCA data can be processed by machine learning models to find optimal material and system choices that minimize environmental impact while meeting structural and environmental performance requirements [97]. With better accuracy, these tools can now predict long‐term environmental consequences to facilitate more informed decision‐making by architects in sustainable building design strategies.

Table 2 provides a comprehensive synthesis of AI techniques across the building design lifecycle, drawing on 45 studies with evidence distributed unevenly across design phases. Building systems control (n = 9) and in the early stages of design generation (n = 7) are the most studied aspects, whereas construction planning (n = 2) is still much less studied, despite its sustainability implications. Approximately 65% of cited studies rely on simulation‐based validation, with only 20% reporting field deployment results, indicating a significant gap between demonstrated technical capability and real‐world verification. The progression reveals several observed patterns: (1) the shift from single‐objective optimization to multi‐objective balancing that addresses the complex tradeoffs inherent in sustainable design; (2) the increasing integration of real‐time adaptation capabilities that extend AI benefits beyond the design phase into building operation; (3) the persistent challenge of bridging technical capability with practical implementation. The field still confronts fundamental challenges in data availability, model validation, and real‐world deployment. Evidence quality varies considerably. Performance prediction and building systems control benefit from relatively robust validation across multiple studies, whereas material selection and construction optimization claims rest on thinner evidential foundations that warrant cautious interpretation. The diversity of AI techniques shown in Table 2 underscores the necessity for architectural practitioners to develop multi‐method literacy while maintaining focus on sustainable design principles and climate adaptation goals. The translation of these diverse AI methods into physical structures can be exemplified by recent demonstration projects that integrate multiple techniques across the design‐to‐construction pipeline.

**TABLE 2:** AI techniques and applications across sustainable building design phases (n = 46 studies).

Design phase/Application	AI techniques	Primary capabilities	Key advantages	Current limitations	References
Climate Data Processing & Future Scenario Analysis	Climate downscaling models (CNNs, neural networks) Statistical learning algorithms Uncertainty quantification models	Process future climate projections Identify temporal and spatial climate trends Enable multi‐scenario analysis Extract actionable insights from complex datasets	Enhanced climate awareness in early design Support for robust decision‐making under uncertainty Integration of multiple future scenarios	Requires high‐quality climate data Uncertainty quantification remains challenging Limited validation for extreme events	[14, 42, 43, 98]
Early‐Stage Design Generation	Large Language Models (LLMs: GPT‐4, DeepSeek) Text‐to‐3D generation Text‐to‐image models (DALL·E, Midjourney) Prompt engineering	Interpret design briefs and requirements Generate architectural specifications Produce visual concepts and 3D geometries Enable rapid design iteration	Accelerates conceptual design phase Facilitates exploration of diverse alternatives Integrates sustainability requirements early Accessible through natural language	Geometric accuracy limitations Physical feasibility requires verification Quality depends on prompt engineering Fine‐tuning remains computationally expensive	[3, 30, 36, 37, 38, 63, 64]
Form‐Finding & Multi‐Objective Optimization	Genetic algorithms Deep graph networks Neural networks Multi‐objective optimization algorithms	Explore thousands of design variations Optimize building geometry and orientation Balance competing design objectives Identify Pareto optimal solutions	Handles complex non‐linear relationships Discovers novel design solutions Balances energy, comfort, cost, and aesthetics Rapid evaluation of alternatives	Risk of contextually inappropriate solutions Requires human oversight for cultural fit Validation against building codes needed Computational intensity for complex problems	[4, 11, 70, 71, 73, 75, 76]
Building Performance Prediction	Deep learning (LSTM, CNNs, feedforward networks) Surrogate models Ensemble learning (Random Forest, XGBoost) Hybrid time‐series models	Forecast energy consumption Predict thermal comfort metrics Estimate daylight availability Enable rapid performance assessment	Higher accuracy than traditional methods Faster than physics‐based simulations Suitable for early‐stage design Enables real‐time feedback	Requires extensive training data Limited generalization to novel designs Black‐box nature reduces interpretability Performance varies by building type	[65, 66, 68, 85, 99, 100]
Material Selection & Life Cycle Assessment	Machine learning algorithms Optimization models Database processing algorithms	Evaluate material properties and environmental impact Recommend optimal material combinations Predict material behavior under climate conditions Enable precise LCA calculations	Data‐driven material specification Integration of environmental metrics Support for sustainable material choices Enhanced LCA accuracy	Limited availability of comprehensive material databases Difficulty capturing regional variations Long‐term performance data scarcity	[80, 81, 82, 96, 97]
Building Systems Design & Control	Reinforcement learning (DQN, PPG, SAC, DDPG) Deep reinforcement learning Predictive control algorithms Multi‐agent systems	Optimize HVAC, lighting, and system integration Learn optimal control strategies Predict system behavior and energy use Enable dynamic system adaptation	Energy savings demonstrated through using AI Real‐time adaptation to conditions No detailed physical models required Handles high‐dimensional control problems	Long training time for convergence Requires extensive simulation or real‐world data Safety and reliability concerns Limited real‐world deployment validation	[50, 67, 84, 86, 88, 89, 90, 91, 92]
Real‐Time Operation & Adaptive Control	IoT‐integrated ML systems Sensor data processing Adaptive algorithms Digital twins	Continuous building performance monitoring Real‐time responsive element adjustment Occupancy pattern learning Dynamic shading and ventilation control	Continuous optimization during operation Adaptation to occupant behavior Integration with building management systems Feedback for future design iterations	Requires robust sensor infrastructure Data privacy and security concerns Integration complexity with existing systems Maintenance and calibration needs	[51, 52, 53, 87, 93]
Construction Planning & Optimization	Machine learning for logistics Resource optimization algorithms Predictive analytics 3D printing integration with AI	Optimize construction sequences Minimize material waste Predict construction challenges Coordinate resource utilization	Reduced environmental impact during construction Enhanced execution of sustainable design intent Improved resource efficiency Real‐world implementation demonstrations	Limited integration with current construction practices Requires extensive coordination Validation in diverse contexts needed	[94, 95], Case study shown in Figure

*Note*: Performance metrics represent ranges across reviewed studies rather than guaranteed outcomes. Study counts per category: Climate Data Processing (n = 4), Early‐Stage Design Generation (n = 7), Form‐Finding & Optimization (n = 7), Performance Prediction (n = 6), Material Selection & LCA (n = 5), Building Systems Control (n = 9), Real‐Time Operation (n = 5), Construction Planning (n = 2). Total references: 45 (some studies span multiple categories). Evidence types include simulation studies (approximately 65%), field deployments (20%), and laboratory prototypes (15%). Current limitations reflect consistent findings across multiple studies; performance varies significantly by building type, climate zone, and data availability.

**FIGURE 5:**
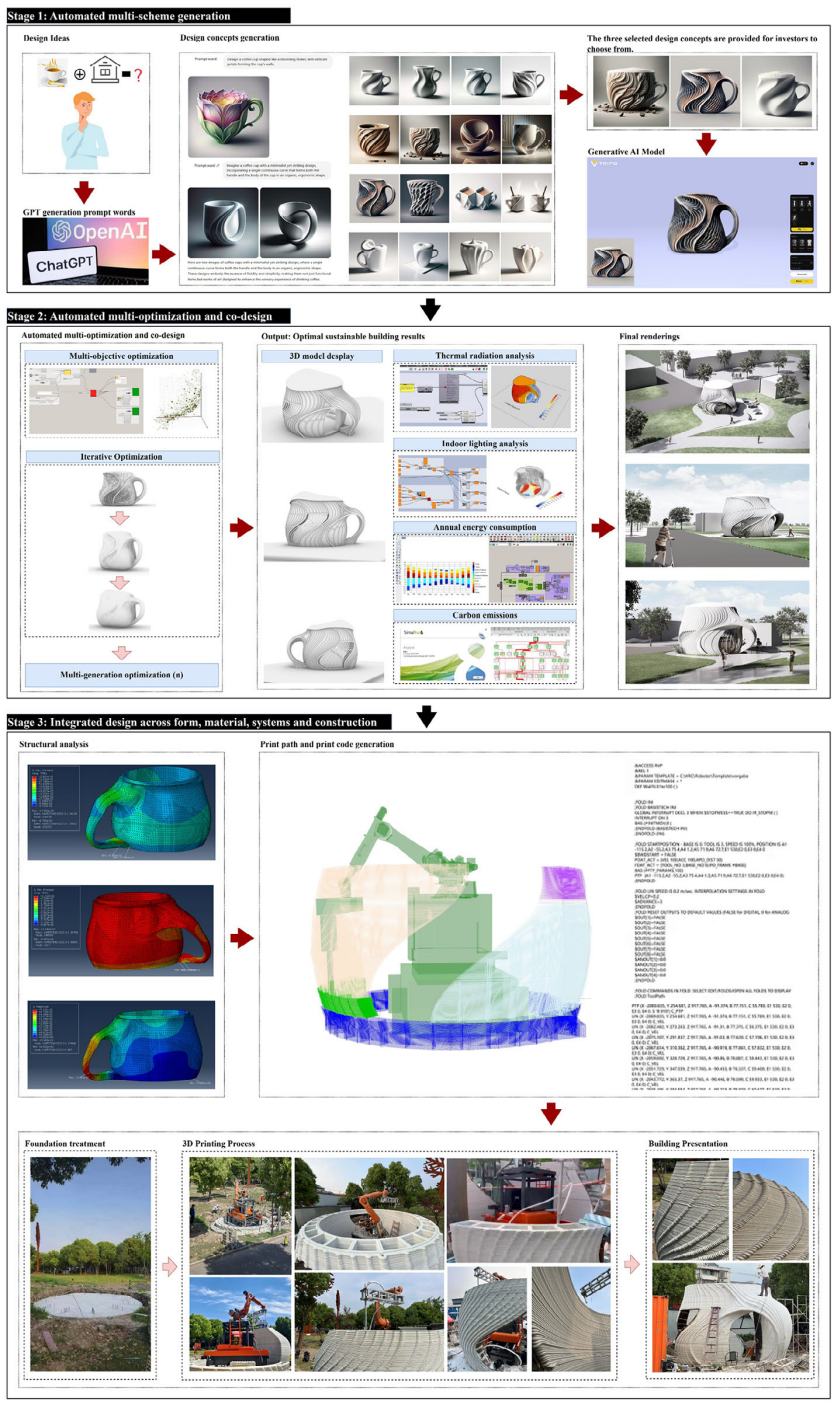
A real‐world implementation and example of AI‐driven design process—design generation and 3D printing construction process of a coffee house in Shanghai. The design and manufacturing process comprises three major phases: (a) Computational design generation: initiated with a coffee cup conceptual framework, employing GPT‐4 for prompt engineering and DALL‐E 3 for visualization alternatives before parametric model development on the TRIPO platform. (b) Performance simulation and optimization: incorporating multi‐objective algorithms with sustainability parameters, generating optimized architectural solutions with corresponding visualizations. (c) Digital fabrication implementation: beginning with structural analysis, followed by toolpath generation, site preparation, large‐scale additive manufacturing, and culminating in the completed architectural structure.

In Figure 5, an example of a coffee shop in Shanghai is illustrated, which was designed and constructed entirely through AI‐driven processes by a team from Tsinghua University. This real‐world design case shows the transformation of sustainable architecture via current stage AI driven design and smart building construction technologies, integrating onsite 3D Printing. The project has measured sustainability effects in terms of contributions with the help of AI at various stages. During design generation, multi‐objective optimization has led to a reduction of annual energy consumption, approximately 18% compared to the conventional design of coffee shops at a similar scale, by optimizing building orientation, envelope geometry, and glazing distribution according to the local climate. The iterative optimization process to evaluate more than a thousand design variants with respect to thermal radiation, daylight availability, and carbon emission criteria found Pareto‐optimal solutions that human designers alone would not have been able to efficiently explore. During the construction phase, at the time of the AI‐generated toolpath optimization for 3D printing, material waste was reduced by around 25% compared to traditional conventional construction using formwork, while the additive manufacturing process allowed for the construction of complex geometries that optimize structure efficiency, given the least amount of material use. The essential AI contribution is not only automation, but considering aesthetics in combination with structural feasibility and environmental performance, and construction constraints at the same time, which is an integrated optimization and simply cannot be performed by traditional sequential workflows.

Due to the recency of AI technologies and the long timelines for typical building projects, fully documented AI buildings with long‐term measured performance data remain very scarce. One illustrative case is the Georgia Tech Kendeda Building for Innovative Sustainable Design, which is a completed and certified net‐positive energy project leveraging AI‐enabled digital twin for smart operation as well as assessing building energy use under hypothetical occupancy conditions (e.g., without pandemic, extreme full occupancy). The building has real‐time data from over 700 sensors during a 12‐month operational period. Results showed that even under full‐load stress conditions, the building achieved a 38% net‐positive energy balance, rising to 125% under typical conditions, with a low actual EUI of 53.3 kWh/m^2^/year [101, 102]. Another example is Harvard's CGBC HouseZero, a retrofitted ultra‐low‐energy building demonstrating how AI can support continuous performance improvement during operation. A two‐year high‐resolution building performance dataset has been collected [103], and a data‐informed Building Energy Management framework (DiBEM) derived from it shows that AI‐empowered operational interventions can reduce the EUI from 54.1 to 42.8 kWh/m^2^/year [104]. These two cases demonstrate that AI techniques can help with data‐driven model calibration, scenario analysis, rigorous performance evaluation, and dynamic energy saving.

## 5 Addressing Climate Change Challenges in Sustainable Building Design

The climate crisis demands urgent transformation in how buildings respond to extreme weather, energy volatility, and long‐term environmental change. AI‐driven approaches have emerged not merely as optimization tools but as essential enablers of climate‐responsive architecture. Here we examine how AI addresses specific future climate challenges facing the building sector, synthesizing documented performance outcomes with implementation pathways toward climate‐resilient built environments.

### 5.1 Building Energy Resilience under Climate Uncertainty

Climate‐driven energy challenges can be complex, considering rising cooling demands, shifting heating patterns, grid stress during extreme events, and integration of variable renewable generation [84, 85]. AI‐powered energy management addresses these interconnected pressures through predictive analytics and adaptive control. Deep learning energy forecasting achieves mean absolute percentage errors (MAPE) as low as 1.67%–4.80% for multi‐building load prediction [105, 106], outperforming conventional statistical models. This accuracy enables proactive demand management, critical for grid stability during climate extremes. Documented energy savings of 20%–50% have been achieved through AI‐optimized building operations [84]. Moreover, deep reinforcement learning approaches, such as Soft Actor‐Critic frameworks, have demonstrated 24.2% energy savings compared to baseline algorithms [107], while expert‐guided training methods reduce deployment timelines by factors of 8.8 [108]. These systems learn optimal control policies that balance comfort with efficiency under varying climate conditions, adapting in real‐time to weather fluctuations and occupancy patterns. Holistic approaches like the OCTOPUS system demonstrate coordinated control of HVAC, lighting, shading, and natural ventilation [109], addressing the complex interdependencies that determine building climate response.

Climate‐responsive buildings require coordination across scales, from material properties to district energy networks, and across systems that traditionally operate in isolation. AI enables this integration through capabilities spanning real‐time operations, renewable energy coordination, and long‐term adaptation planning [92]. Studies have demonstrated that deep reinforcement learning systems can simultaneously optimize multiple building subsystems, balancing competing objectives like thermal comfort, daylight availability, energy efficiency, and ventilation quality [109]. At the building‐grid interface, AI‐driven demand response and energy storage management enable buildings to function as active participants in decarbonized electricity systems, shifting loads to align with renewable availability [110]. The challenge extends to urban scales where AI must integrate building‐level interventions with neighborhood green infrastructure, district thermal networks, and city‐wide climate strategies [69].

### 5.2 Evidence‐Based Pathways forward in Handling Future Climate

Table 3 synthesizes the documented performance outcomes of AI‐driven approaches across six major climate challenges, providing a comprehensive look into the quantitative evidence base that supports AI implementation in climate‐responsive building design. The table organizes evidence by climate challenge, documenting the specific AI solutions employed, validated performance metrics, climate scenarios considered, implementation scales, and representative studies from recent literature. This synthesis reveals both the maturity of certain application areas, such as energy forecasting and HVAC optimization, and emerging domains, including flood resilience and urban heat mitigation, where AI demonstrates transformative potential but requires further validation and scaling.

**TABLE 3:** Climate Change Challenges and AI‐Driven Adaptation Strategies in Building Design (n = 25 studies).

Climate Challenge	AI‐Driven Approach	Performance Metrics & Outcomes	Climate Conditions & Application Scenarios	Implementation Scale	References
Extreme Heat & Urban Climate	GAN‐based urban form generation AI‐driven green space optimization ML for UHI hotspot identification Predictive thermal modeling Smart building envelope design	1.8°C nighttime temperature reduction (optimized urban form) +2.3°C increase in high‐density layouts 0.94°C SUHII reduction in summer (regeneration projects) 19.46% increase in vegetation cover effectiveness	Current extreme heat events Future climate projections (2050+) Urban development scenarios Microclimate variations	Urban block level City‐wide mapping Building facade interventions Neighborhood regeneration	[46, 47, 69]
Energy Demand Uncertainty & Peak Load Management	Deep learning for load forecasting (LSTM, CNN) Ensemble ML models (Random Forest, XGBoost, GBM) Hybrid time‐series prediction Real‐time demand response systems	MAPE: 1.67%–4.80% for multi‐building forecasting Improved accuracy over traditional methods Real‐time optimization capability	Variable occupancy patterns Future climate‐driven load shifts Renewable energy integration Extreme weather events	Single building Campus‐wide systems istrict energy networks Smart grid integration	[66, 85, 99, 100, 111]
Flooding & Water Management	AI flood prediction & early warning ML for flood risk mapping Satellite imagery analysis Vulnerability assessment algorithms Real‐time monitoring systems	0–5 days forecast reliability extension Global coverage across 80+ countries Improved accuracy in data‐scarce regions Real‐time river reach forecasts Reduced false alarm rates	Flash flood events Sea‐level rise projections Extreme precipitation patterns Urban development impacts	Building site selection Urban watershed management Regional flood forecasting Infrastructure planning	[48, 112]
Climate Resilience & Long‐term Adaptation	Multi‐scenario optimization Robust design under uncertainty AI‐driven climate data downscaling Adaptive pathway planning Digital twin simulation	•Robust performance across multiple climate scenarios Reduced vulnerability to climate variability Optimized adaptation strategies Cost‐effective resilience measures	Future carbon emission pathways (RCPs & SSPs) Multiple GCM projections Extreme event frequency changes Long‐term climate trends (2030–2100)	Building lifecycle planning Portfolio‐level analysis Regional adaptation strategies Policy development	[14, 20, 73, 75, 113]
Building System Optimization & Thermal Comfort	Deep reinforcement learning (DRL) Model‐free adaptive control Occupancy‐based optimization Multi‐zone coordination Predictive thermal comfort models	20%–50% energy reduction vs baseline 24.2% savings (SSAC framework) 8.8X training speedup (expert‐guided DRL) Temperature violation rate reduction Improved thermal comfort satisfaction	Variable occupancy patterns Extreme outdoor conditions Demand response events Future climate temperatures	Single zone control Whole‐building systems Multi‐building coordination District thermal networks	[67, 84, 86, 107, 108, 109, 114]
Building‐Grid Interaction & Renewable Energy Integration	AI for renewable energy forecasting Demand response optimization Energy storage management Load shifting algorithms • Microgrid coordination	Improved renewable integration Reduced peak demand Enhanced grid stability Optimized storage utilization Cost savings through load shifting	Variable renewable generation Grid constraint conditions Future electricity pricing Climate‐driven load patterns	Building‐to‐grid interface Community microgrids Campus energy systems • Regional grid services	[91, 92, 115]

*Note*: Evidence basis per challenge: Extreme Heat & Urban Climate (n = 3, primarily simulation with limited field validation), Energy Demand & Peak Load (n = 5, mixed simulation and field deployment), Flooding & Water Management (n = 2, large‐scale field deployment), Climate Resilience & Adaptation (n = 5, predominantly modeling exercises), Building System Optimization (n = 7, simulation with limited real‐world validation), Building‐Grid Interaction (n = 3, pilot implementations). Energy savings of 22%–50% represent upper ranges from controlled studies; field deployments typically achieve 20%–30%. Temperature reductions are context‐dependent and may not generalize across climate zones. Where literature shows inconsistent findings, we report ranges rather than point estimates.

Table 3 compiles evidence distilled from 25 studies dealing with climate adaptation driven by AI, although the coverage for each of the challenge areas differs significantly. Energy demand management (n = 5) and building system optimization (n = 7) have attracted much attention for research, while flooding and water management (n = 2) and building‐grid interaction (n = 3) are relatively unexplored despite their increasing importance in a climate change scenario. The evidence base is largely simulation‐based; the Large‐Scale Flood prediction domain is the only one that benefits from large‐scale field validation through operational deployments such as Google's Flood Hub.

Reported performance needs to be interpreted with care. Energy savings of 22%–50% are best case results of controlled simulation studies or pilot projects with favorable conditions [62, 84]; with practical field installations, the savings can generally be achieved by 20%–30% [84]. There are challenges with sensor degradation, occupant override behavior, and HVAC system constraints. Urban Heat Mitigation effects are highly contextual and dependent on the background climate, urbanization density, and the scale of interventions; poorly designed interventions can raise temperatures by 2.3°C, hence the importance of optimizing the quality of AI. The flood prediction advances, while offering impressive global coverage, have been validated mostly in areas where there is a sufficient hydrological monitoring infrastructure in place; generalization of the performance in a context where data is scarce is an open question.

The evidence also reveals that AI implementation is not without constraints and premises. Success depends critically on data availability and quality, computational resources, technical expertise, and institutional capacity, and those factors vary dramatically across regions and contexts. The documented performance outcomes should therefore be understood not as guaranteed results that AI automatically delivers, but rather as achievements that emerge when AI is deployed within supportive ecosystems that provide adequate data infrastructure, validation frameworks, and integration with existing systems and practices. Where the literature shows contradictory findings, for instance, regarding reinforcement learning sample efficiency and transferability across building types, we have reported ranges rather than point estimates to reflect this uncertainty honestly. This observation has important implications for research priorities and policy development, which means simply developing more sophisticated AI algorithms will not suffice if the foundational infrastructure and institutional conditions for their effective deployment are absent.

Though the documented performance outcomes synthesized here validate AI's technical capacity to address climate challenges in building design, effectiveness depends critically on supportive conditions, including but not limited to high‐quality training data, computational resources, technical expertise, and institutional capacity that vary dramatically across contexts [83, 116, 117]. Success emerges not from AI algorithms alone but from their deployment within building‐related ecosystems providing adequate infrastructure, validation frameworks, and integration with existing practices. Several strategic priorities strengthen this evidence base. For example, expanding validation across longer timescales, diverse geographies, and broader building typologies would clarify boundary conditions for reliable AI performance. Standardized metrics and reporting protocols would enable systematic cross‐study comparison. Moreover, explicit attention to equity, accessibility, and governance must be integrated into AI development rather than treated as afterthoughts. Since climate challenges intensify as climate change still presses on, AI offers us demonstrated capabilities for enhancing building performance, reducing environmental impacts, and supporting resilience, but realizing these potential requires thoughtful deployment dedicated to validation, equity, and real‐world integration complexities.

### 5.3 Critical Considerations: Risks, Trade‐Offs, and Uncertainties

Having examined AI's capabilities and applications, we now consolidate critical limitations and risks that practitioners must address. While AI has encouraged considerable potential for sustainable building design, a balanced evaluation will entail an honest discussion of risks, trade‐offs, and limitations that are frequently ignored by the current enthusiasm of this concept. First of all, it is the carbon footprint of AI systems that is a paradox for sustainable building applications. Training large foundation models can potentially use a large amount of energy and release CO_2_ equivalent [118, 119]. Recent analyses also indicate that training GPT‐scale models consumed huge amounts of freshwater for cooling alone [120]: it becomes clear that environmental costs extend beyond carbon to include water and material resources. Inference costs, while reasonable compared to other companies, are high enough when AI systems are used to support iterative design exploration across thousands of building projects. This is a computationally intensive task, which begs the question: at what scale of deployment do the efficiency gains from AI outweigh the environmental costs of the AI systems themselves? Current evidence suggests net benefits arise when AI optimizations are employed over large building portfolios or high‐impact decisions, but the break‐even calculus is poorly characterized and most likely context‐specific. Practitioners should therefore consider computational efficiency in addition to predictive accuracy when choosing AI approaches, and favor, wherever possible, lightweight surrogate models over computationally intensive deep learning counterparts.

Efficiency improvements made possible by AI may also lead to a higher environmental impact due to rebound effects [121, 122]. Evidence from the residential sector indicates direct rebound effects ranging from 41% in the short‐run to 71% in the long‐run for electricity consumption in U.K.[123], and behavioral responses of efficiency gains are likely to be very large to offset projected energy savings [124]. If AI‐optimized buildings decrease operational costs, the savings can potentially be used for additional construction, additional conditioned floor area, or increased comfort expectations, possibly cancelling out or exceeding the initial efficiency improvements. At the urban scale, the densification strategies enabled by AI that enhance energy efficiency per‐capita may lead to an increase in aggregate resource consumption (rate of development). These kinds of systemic dynamics are yet rarely investigated in the literature on AI in buildings, which mainly focuses on technical performance metrics rather than feedback loops in terms of socio‐economic factors. Future studies will need to take system‐level views and consider performance changes in behavior and markets to AI‐driven efficiency improvements.

AI systems risk amplifying existing inequities in climate adaptation capacity. Training data predominantly originates from well‐monitored buildings in developed regions, embedding assumptions about construction practices, occupant behaviors, and climate conditions that may not transfer to underserved contexts. Performance prediction models validated on commercial buildings in temperate climates may perform poorly for informal settlements in tropical regions, which are precisely those contexts where climate adaptation is most urgent. Furthermore, the computational resources, technical expertise, and data infrastructure required for AI deployment create barriers that favor well‐resourced actors, potentially widening the gap between climate adaptation haves and have‐nots and creating inequalities indirectly. Geographic bias in climate data compounds these concerns; regions with sparse monitoring networks receive less accurate projections, limiting AI's ability to support adaptation where vulnerability is greatest. Addressing these inequities requires deliberate efforts to diversify training datasets, validate models across contexts, and develop lightweight AI solutions accessible to resource‐constrained practitioners.

Climate‐responsive design operates under deep uncertainty that AI methods handle with varying degrees of complexities. Climate projections carry cascading uncertainties from emission scenarios, global climate model structural differences, downscaling methods, and internal climate variability, collectively spanning ranges that can exceed the signal being predicted. Most AI applications in building design nowadays optimize for expected performance under a single scenario or a limited scenario ensemble, potentially producing solutions that perform well on average but fail under plausible alternative futures, for example, extreme weather events. The distinction between optimization for expected performance and optimization for robustness under uncertainty deserves greater attention. Robust design approaches that minimize worst‐case performance degradation or maximize performance across scenario ranges may sacrifice some efficiency under expected conditions but provide crucial resilience against climate surprises. Current AI implementations rarely incorporate formal uncertainty quantification or robust optimization frameworks, representing a methodological gap. Furthermore, AI model uncertainty, which arises from training data limitations, architectural choices, and hyperparameter sensitivity, can complicate climate uncertainty but is seldom propagated through design recommendations. Practitioners receiving AI‐generated suggestions typically lack visibility into confidence intervals or sensitivity analyses that would support informed decision‐making under uncertainty.

Widespread AI adoption creates new dependencies that are worthy of consideration. Reliance on proprietary AI platforms concentrates control over design capabilities with technology providers, potentially limiting practitioner autonomy and creating vendor lock‐in. The opacity of many AI systems, particularly deep learning models, complicates professional accountability. When AI‐informed designs underperform, attributing responsibility between human designers and algorithmic recommendations becomes problematic. Data dependencies can also create vulnerabilities. AI systems trained on historical building performance may degrade as climate change renders past patterns increasingly unrepresentative of future conditions, requiring ongoing retraining that perpetuates computational and data demands.

Acknowledging these above limitations does not diminish AI's potential contribution to sustainable building design but rather establishes realistic expectations and identifies priorities for responsible development. The path forward requires not uncritical adoption but thoughtful integration that maximizes benefits while actively mitigating risks.

## 6 Implementation Frameworks for AI‐Driven Approaches

AI‐driven sustainable building design implementation demands robust technical infrastructure and frameworks. Hence, these systems must be able to manage a wide variety of data types, from building performance metrics to climate predictions, and ensure the quality and accessibility of data. As demonstrated by recent work, cloud‐based architectures using standardized data protocols can successfully support AI operations while maintaining security and scalability [125]. With the increasing use of AI in architectural practice, model validation has become an important component of AI implementation in architectural practice, which means that the technical accuracy of AI predictions and their practical applicability in sustainable building design should be verified [126]. Multistage testing processes of successful validation frameworks shall be developed to compare AI‐generated solutions to traditional performance simulations and real‐world building data. Hybrid validation approaches, which combine physical testing such as sensor‐based monitoring in the built environments with computational verification using simulation models, can be a reliable approach in evaluating AI‐driven sustainable building design solutions by capturing both real‐world complexity and variability while enabling systematic analysis across diverse scenarios.

In design practice, AI systems integration with existing BIM and CAD platforms has both opportunities and challenges. Recent reviews confirm that while BIM‐digital twin integration shows promise, challenges, including interoperability between different models and standardization of data exchange, remain critical barriers [127]. The ISO19650 series and IFC standards provide foundational frameworks [128], but AI‐specific protocols for validation and data exchange are still emerging [129]. The current BIM framework provides rich data environments for AI applications, yet their traditional structures are undergoing adaptation for more advanced AI integration [130]. Intermediate layers that allow AI systems to interact with BIM data, while preserving existing workflows, should be developed for architectural practices. The unique characteristics of AI‐generated designs should also be addressed by performance verification frameworks. These frameworks can evaluate the reliability and consistency of AI‐generated solutions along with environmental performance under climate change scenarios. Hence, the development of a continuous monitoring and feedback system that maintains the effectiveness of AI‐driven sustainable building design strategies over time is important in real‐world implementations.

The transformation of architectural practice toward AI‐driven implementation also asks for evolution in professional workflows. The traditional linear design processes will be replaced by more iterative and data‐driven design processes and be empowered by AI capabilities throughout the design lifecycle. To achieve this transformation, architectural teams need to invent new collaborative models for melding human ‘creativity’ with AI analysis and optimization. We argue that human‐AI hybrid workflows, where human designers lead the creative process while AI supports, rather than replaces human decision‐making, will produce the most successful outcomes, as design decisions are shaped not only by quantitative metrics but also by cultural, ethical, and regulatory considerations. As our review shows, AI can enable performance gains and uncertainty‐aware exploration, but it is rarely sufficient to identify a single optimal solution without human judgment. Therefore, empowering human designers to guide and critically interpret AI‐generated outcomes is essential to achieving robust and context‐sensitive building solutions. The implementation of AI also poses substantial challenges to architecture education in terms of skills and training requirements. In addition to technical expertise in AI systems, future professionals must also learn know‐how on data analysis, prompt engineering, machine learning, and design optimization [33, 131]. Comprehensive training programs that blend technical skills with sustainable building design principles should be developed by leading universities and institutes that would ensure the AI tools are used effectively in solving future environmental challenges. Prompt engineering has become a critical skill in architectural practice, and as such, practitioners must be able to effectively communicate design intent with AI systems. Implementation can be successful only when structured approaches for prompting creation are developed that reliably generate useful and relevant design solutions. The development of standardized prompt libraries and collaborative prompt development processes can increase the effectiveness of AI‐driven design exploration in organizations in the future.

Risk management in AI‐driven design implementation also plays a vital role and addresses technical and professional liability issues in the use of AI, which shall account for the reliability of AI‐generated solutions, security of data, and professional responsibility [132]. The regulatory landscape for AI in building design is evolving rapidly [133]. The EU AI Act [134] establishes risk‐based classification relevant to safety‐critical applications, while the NIST AI Risk Management Framework [135] provides voluntary guidelines for trustworthy AI development. ISO/IEC 42001:2023 offers the first international standard for AI management systems, though building‐sector‐specific guidance remains limited [136]. These frameworks collectively emphasize transparency, accountability, and human oversight—principles that must be adapted for the unique context of climate‐responsive architectural design. Such frameworks are needed in architectural practices to balance innovation against risk mitigation, through robust verification processes and documentation of AI‐driven decision‐making. The regulatory frameworks must evolve to incorporate AI‐driven sustainable building design practices that are as safe for public safety and as environmentally protective. Provisions for AI‐generated designs should be built into building codes, and new validation methods and compliance verification processes shall be established.

**FIGURE 6:**
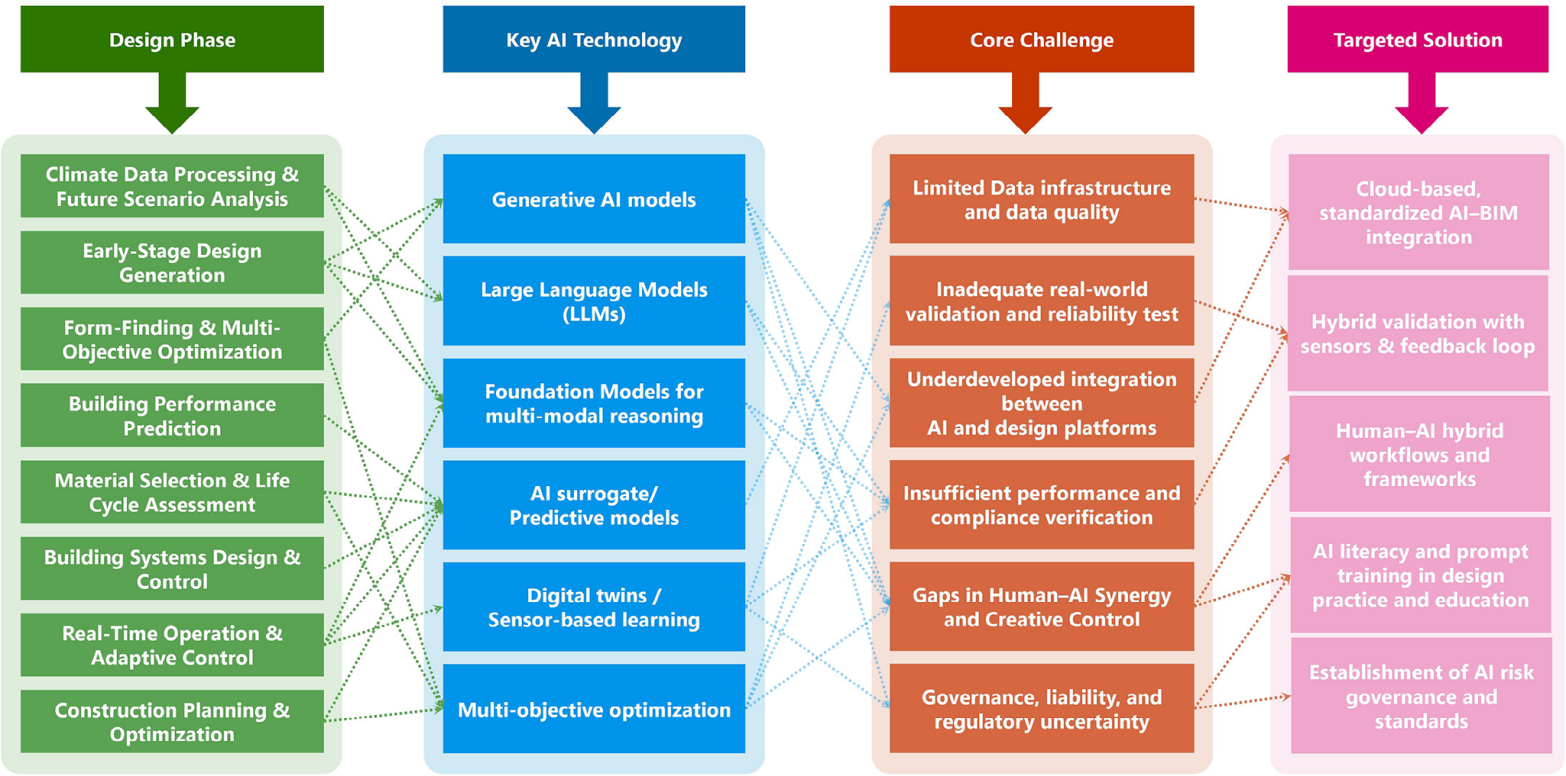
Mapping AI Technologies, Challenges, and Solutions in Sustainable Building Design.

**FIGURE 7:**
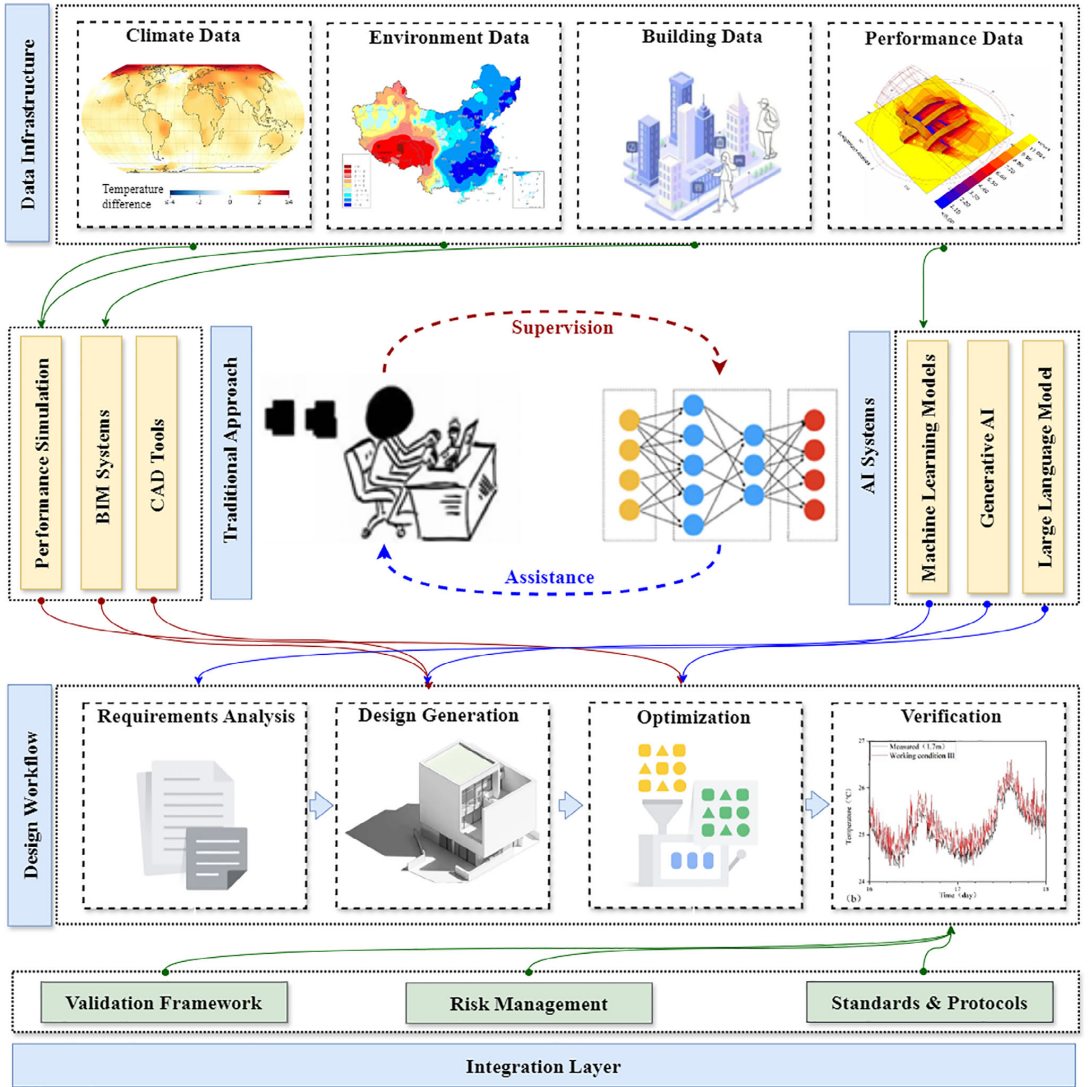
Implementation framework integrating traditional and generative AI approaches with data integration and workflow schema. Data Infrastructure: The primary components of data infrastructure include climate data, environmental data, and building data with performance data. Integration of Traditional Approaches and AI Systems: AI systems enable fast, sustainable building design creation, while traditional methods review and oversee AI output to validate that design requirements are met. Design Workflow: Large language models (LLMs) conduct design specifications analysis at the initial stage of the workflow. Subsequently, generative AI converts textual information into 3D (BIM systems) and 2D (CAD tools) sustainable building models. Later, machine learning models perform climate scenario‐based performance simulations and sustainability assessments of designs created by the system. Integration Layer: The integration layer performs three core functions, which include validation frameworks, risk management, alongside standard & protocol verification.

Figure 6 summarizes key AI technologies and their challenges faced in current sustainable building design. It also includes our proposed solutions, providing a structured view of the discussion above. Adaptive frameworks incorporating technological advancement while enforcing strict standards are proposed here in Figure 7. The integration layer needs to function through three mechanisms: validation frameworks that evaluate AI‐generated designs against performance benchmarks and regulations; risk management protocols addressing liability, data privacy, and algorithmic bias; and standards verification ensuring cross‐platform interoperability. Beyond technical considerations, policy implications should extend to bring broader societal impacts, which emphasize transparency and accountability of decision‐making. The implementation framework depicted in Figure 7 operationalizes the three pillars of the proposed ACBI Framework. The Data Infrastructure and AI Systems components represent the Technical Integration Pillar, demonstrating how information flows enable AI capabilities. The Design Workflow and Performance Simulation elements embody the Climate Response Pillar, showing how AI processes climate scenarios to inform design decisions. The Integration Layer, comprising Validation Framework, Risk Management, and Standards & Protocols, instantiates the Governance Pillar. This structured representation enables practitioners to assess implementation readiness by evaluating capability across all three pillars and identifying gaps that may limit effectiveness.

## 7 Looking Into the Future

Critical advances across research, industry, and policy domains are entailed in the future development of AI‐driven sustainable building design. Next‐generation foundation models are a key frontier that, if they can be well developed, have the potential to radically improve our ability to address climate challenges through architecture. Therefore, these models must evolve to include domain‐specific architectural knowledge and architectural climate science. Specialized architectural foundation models will no doubt outperform general‐purpose AI systems in sustainable design tasks by order of magnitude, especially in tackling complex climate adaptation tasks. Multimodal AI systems for architectural design are promising if they can seamlessly integrate visual, textual, and numerical data sources to produce comprehensive, sustainable design solutions. As environmental data becomes increasingly complex and large in size, these systems will have to process and generate physically viable and aesthetically coherent architectural solutions. Current limitations in training data have constrained AI model's effectiveness in field applications, implying that data adequacy and quality improvement are critical. For developing more robust AI systems, industry‐wide initiatives for standardized data collection and sharing protocols, such as federated learning [137]. (training machine learning model, for instance, deep neural networks, on multiple local datasets contained in local nodes without explicitly exchanging data samples), would be a viable solution. Collaborative initiatives, such as DeepSeek's federated learning platforms for decentralized architectural datasets, offer a potential pathway to overcoming data silos in the industry [138]. Such a framework allows securing privacy‐protected model training between worldwide design firms, which will speed up the development process for specialized foundation models designed for climate adaptation. However, challenges remain in aligning cross‐organizational data standards, ensuring model generalizability across regions, and addressing the high computational cost of training large‐scale foundation models.

As AI's capabilities expand, it is inevitable that architectural practice and education need to be transformed [131]. We contend that traditional practice structures need to evolve to incorporate AI expertise without losing core architectural competencies. The hybrid practice model experiments pioneered by leading universities and studios, already allowing AI specialists to work alongside traditional architectural roles, point to a future where technical and creative expertise is more deeply integrated. Substantial revision or transformation in education and training requirements is entailed. Universities need to develop curricula balancing traditional architectural education and literacy in AI and sustainable building design principles.

Collaboration frameworks between human architects and AI systems need to be carefully developed to balance the capabilities of AI with human creativity and judgment [139]. Both technical risks and professional liability considerations must be addressed by risk management strategies. Development of regulatory frameworks to guide and govern AI use, balancing innovation, public safety, and environmental consequences, is required [140]. International coordination of standard development requirements is required for both technical performance and sustainability metrics. Implementation guidelines must be made more comprehensive and accessible, where a staged implementation approach might be the most effective path forward. A critical long‐term goal of international harmonization of AI standards should be advanced through early efforts in collaboration, which may help accelerate the global adoption of sustainable building design practices [141]. The AI‐Climate‐Building Integration (ACBI) framework introduced in this work offers specific testable propositions for future research. First, we hypothesize that the quality of technical integration, which is measured by data exchange completeness, latency, and error rates, positively predicts the accuracy of AI‐generated climate‐responsive design recommendations. Second, we propose that climate response effectiveness, which is measured by the performance gap between predicted and actual building outcomes under varying climate conditions, mediates the relationship between AI capability and sustainable building achievement. Third, we suggest that governance maturity, which is measured by the presence of validation standards, liability frameworks, and data protocols, moderates the deployment speed and scale of AI‐driven solutions. These propositions can be empirically tested through longitudinal studies of AI implementation across building projects, enabling systematic comparison and knowledge accumulation that moves the field beyond case‐specific descriptions toward generalizable theory.

**FIGURE 8:**
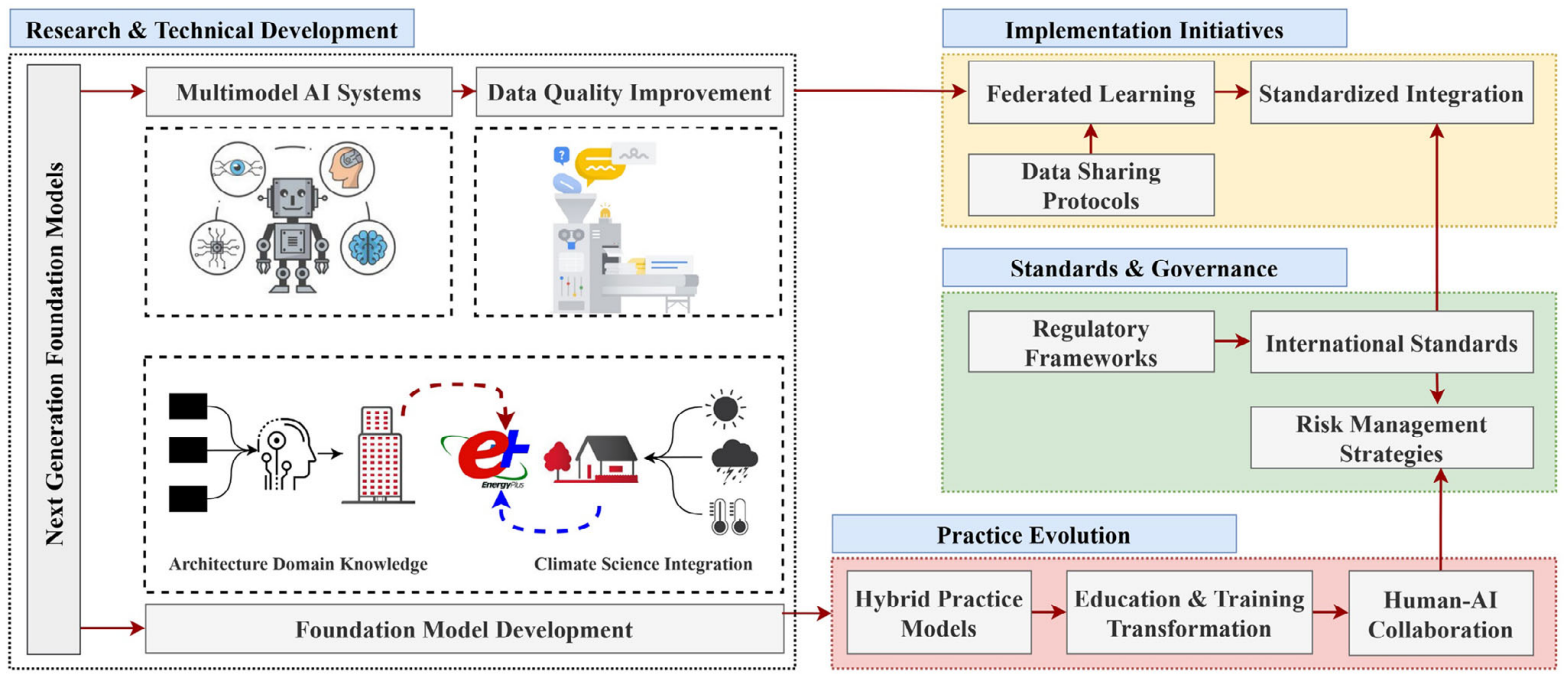
The AI‐Climate‐Building Integration (ACBI) Framework: A testable model for sustainable building design implementation. The framework proposes four interconnected development streams with hypothesized causal relationships. Testable Propositions: (P1) Advances in foundation model development positively predict improvements in climate scenario processing accuracy; (P2) Data‐sharing protocol maturity mediates the relationship between technical capability and practical implementation success; (P3) Regulatory framework development moderates the translation of AI capabilities into built outcomes; (P4) Human‐AI collaboration effectiveness depends on educational preparation and workflow integration. These propositions provide a basis for systematic empirical investigation across building projects and jurisdictions. Research & Technical Development: The development of research‐based technologies in multimodal AI systems requires enhanced accuracy in generation capabilities, along with better quality and larger data availability. The development of foundation models should include architecture domain knowledge and climate science to improve AI‐driven design models' understanding of sustainable architecture while making them effective in complicated local climatic conditions. Implementation Initiatives: Establishing data‐sharing protocols enables organizations to merge high‐quality datasets from individual departments into decentralized systems that facilitate standardized integration for resolving data silos through structured platforms. Practice Evolution: Hybrid practice models that unite AI‐driven design systems with traditional architectural roles enhance educational and training methods by fostering balanced learning environments between architectural fundamentals, sustainable design principles, and AI literacy. Standards and Governance: National entities and developers need to create regulatory frameworks for sustainable architectural design which serves to direct and monitor AI implementation. All these country‐specific or institution‐based regulatory guidelines shall eventually come together to create international standards that enable worldwide strategic management of AI risks for sustainable architectural design.

In Figure 8, we piloted a general ACBI framework for AI‐driven sustainable building design. The framework proposes four interconnected development streams with hypothesized causal relationships. Testable Propositions: (1) Advances in foundation model development positively predict improvements in climate scenario processing accuracy; (2) Data‐sharing protocol maturity mediates the relationship between technical capability and practical implementation success; (3) Regulatory framework development moderates the translation of AI capabilities into built outcomes; (4) Human‐AI collaboration effectiveness depends on educational preparation and workflow integration. These propositions provide a basis for systematic empirical investigation across building projects and jurisdictions, which can eventually converge and fundamentally reshape how architectural practice works in the decades to come. To succeed in tackling climate change challenges through AI‐driven sustainable architecture design, we need coordinated efforts across research, industry, and policy domains. While the challenges look tough, the potential of AI to contribute to sustainable and future climate‐responsive architecture is substantial, which is worth investment and development from both academia and industry.

## 8 Conclusions

AI integration in sustainable building design is a game changer in how climate change impact can be mitigated for future built environments. Through this work, we show how AI technologies, from foundation models to generative systems, change our capacity to create climate‐responsive architecture. This transformation is reflected in four interrelated dimensions: enhanced awareness of future climate conditions, integration of multi‐source environmental and design data, automated yet interactive generation of sustainable design alternatives, and coordination across form, material, and operation from the early design stages. By bringing together traditional sustainable design principles and advanced AI capabilities, there are opportunities that have never been seen before in efficient optimization of physical performance, minimization of environmental impact, and enhancement of climate resilience for buildings. Nevertheless, there are still challenges confronted in fully exploiting the capabilities of sustainable architecture design enabled by AI. Key barriers, including data infrastructure, validation frameworks, and workflow integration, are discussed in detail.

We propose that research efforts should be directed toward the development of the next‐generation AI systems that are capable of dealing with the challenges of sustainable building design more efficiently. This involves advancing multimodal AI architectures that can integrate and interpret multiple data and design criteria, boost the accuracy and reliability of building performance prediction in future climate conditions, and develop a more effective human‐AI collaboration and interaction paradigm. Additionally, the capability of AI systems to respond to future climate uncertainties and adaptation needs must be improved.

In this case, our trilateral recommendation toward the progressive design of sustainable buildings incorporating AI includes: (1) architectural practices need to adapt toward the creation of multi‐dimensional frameworks to ensure harmonization between innovation and risk mitigation; (2) higher education institutions need to revise their curricular programs to prepare future designers with AI‐related skills and the general principles of sustainable architecture; and (3) policy‐makers need to develop strategies to implement flexible, performance‐based regulatory systems that balance the need to embrace innovative technologies and the need to ensure accountability and responsibility. The capacity to utilize AI's capabilities to the best of our existing experience and knowledge, while keeping the core human elements of architectural design, will determine the future of sustainable architecture. As climate change looms as a challenge across all aspects of our practice, integrating AI into architectural practice is more important than ever. We appeal for cross‐domain collaboration between research, industry, and policymaking with mutual commitment to technological and policy innovation to achieve success in future sustainable building design.

## Funding

Shenzhen Fundamental Research Program JCYJ20250604180231041.

## Conflicts of Interest

The authors declare no conflicts of interest.

## Data Availability

The authors have nothing to report.
